# Investigations of Anti‐Reflux Formulations Containing Alginates Using MRI: A Feasibility Study Using Conventional 3.0T and 0.5T Open Upright Scanning

**DOI:** 10.1002/nbm.70090

**Published:** 2025-07-10

**Authors:** Caroline L. Hoad, Matthias Knarr, Abdulsalam Aliyu, Olivier Mougin, Jan Alappadan Paul, Luca Marciani

**Affiliations:** ^1^ Sir Peter Mansfield Imaging Centre, School of Physics and Astronomy University of Nottingham Nottingham UK; ^2^ NIHR Nottingham Biomedical Research Centre, Nottingham University Hospitals NHS Trust and University of Nottingham Nottingham UK; ^3^ PS Biopolymer GmbH & Co. KG Germany; ^4^ Nottingham Digestive Diseases Centre, School of Medicine University of Nottingham UK

**Keywords:** alginate, anti‐reflux, gastric, MRI, raft strength

## Abstract

Sodium alginates are widely used for their gelling, thickening, and stabilizing properties. Raft‐producing formulations have been used widely for many years to treat the symptoms of anti‐reflux disease and those suffering occasional symptoms. The aim of this study was to determine the feasibility of characterizing rafts formed from alginate‐containing anti‐reflux formulations in vivo using either a supine high‐field 3T scanner or an upright low‐field 0.5T MRI scanner. Six healthy participants (one male, five female, age range 23–50 years) attended three study visits following an overnight fast and were scanned at one field strength (*N* = 3 at 0.5T, N = 3 at 3T) before and after ingestion of an acidic drink followed by one of three different alginate antireflux formulations. These formulations had identical quantities (by mass) of sodium alginate, calcium carbonate, and sodium bicarbonate. Raft position and volume were measured along with gastric contents and gas using T2‐weighted MRI. Additionally, the image textures of the raft and T2 properties were investigated at 3T. In vitro properties (raft strength, mass, and NMR chemical analysis) of the three different formulations were also determined. Alginate rafts were generated in all volunteers for all formulations. Gastric emptying of the acidic drink was consistent across all study days. Raft volumes measured showed some differences between body positions, with upright maintaining a higher volume of raft for longer. In vitro analysis showed significant differences in strength and mass between two of the formulations, which were likely caused by differences in the chemical structure of the alginates used in the formulations. In conclusion, characterization of anti‐reflux alginate raft properties can be achieved using both low‐field upright and high‐field supine MRI. Larger scale studies are needed to determine the differences between formulations in vivo.

## Graphical abstract

MRI was used to visualize rafts generated from anti‐reflux formulations containing alginates in vivo in both supine and upright body positions. The evolution of the raft volumes may differ depending on whether participants sit up or lie down after dosing. In vitro characterization of the rafts from the three formulations studied showed different raft strength due to differences in the chemical composition of the alginates used, despite identical amounts of sodium alginate, calcium carbonate, and sodium bicarbonate in the formulations.

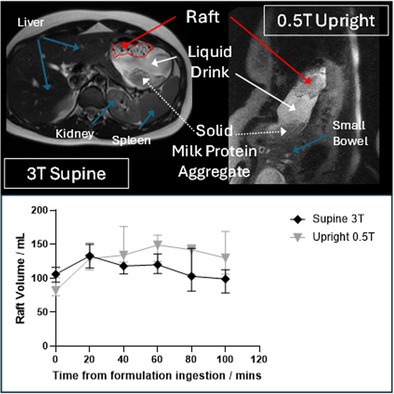

**Abbreviations**
F_G_
: fraction of single guluronate units
F_GG_
: fraction of two G unit block
F_GGG_
: fraction of block containing only three G units in a row
F_GGM_
: fraction of block containing two G units connected to one M unit
F_GM_
: fraction of mixed GM unit block
F_M_
: fraction of single mannuronate units
F_MGM_
: fraction of block containing alternating MGM units
F_MM_
: fraction of two M unit block
FW: Forward
G: Guluronic
M: Mannuronic

## 1 Introduction

Sodium alginate is derived from brown seaweed and belongs to a family of linear co‐polymers containing 1,4‐β‐D mannuronic (M) and α‐l‐guluronic (G) acids. Their physical properties depend on the combination of M and G blocks and vary substantially between the different seaweeds harvested, and also the location within the plants where the alginate is extracted. Alginates have been extensively researched and are widely used for their gelling, thickening, and stabilizing properties, particularly in the food industry [1]. Alginate gels form in the presence of calcium and when the pH of the solution falls below the pKa of the uronic acid groups [2].

Raft producing alginate formulations have been used for many years to treat gastroesophageal reflux disease (GERD) [3] and are also used by those suffering occasional gastric reflux symptoms. The three main ingredients in the formulations are sodium alginate, sodium bicarbonate and calcium carbonate. In the acidic environment of the stomach the CaCO_3_, which is insoluble at neutral pH, is solubilized, the free Ca^2+^ crosslinks the alginate and forms a raft mass. In the acid environment of the stomach parts of the alginates could be protonated on initial contact with the acid, which converts these to a swollen state. The CO_2_ which is released from the carbonate sources helps floating this mass, creating the buoyancy to make the raft float. The formulation forms a physical barrier floating on the surface of the stomach contents and trapping and neutralising the acidic contents away from the gastroesophageal junction preventing damage to the esophageal mucosa [3, 4]. Patients suffering with GERD often have an ‘acid pocket’ sitting next to the esophageal junction following intake of food and the strong alginate rafts can cap the top reducing, neutralising and/or preventing the acid reflux postprandially [5, 6, 7].

Understanding how alginate formulations create raft barriers in the stomach is important for the product design, dose and usage. Magnetic resonance imaging (MRI) can visualise these rafts in vivo and characterise their properties [8, 9] non‐invasively. MRI has been used to monitor the strength of alginate gels through the proton NMR transverse relaxation time T_2_ parameter both in vitro and in vivo [10, 11]. The parameter reflects the mobility of the water protons and strong gels which immobilise the water to a high degree have low T_2_ values where weaker gels have higher T_2_ values.

Conventional MRI scanners have their main magnetic field aligned horizontally along the ‘tunnel’, and participants can only be scanned in the supine, prone or side positions. This is not the general body position of a patient when they consume an oral alginate formulation to generate a gel raft to prevent gastric reflux, although some patients do take them before bedtime. Open MRI systems can be used to study participants in a more natural body position. The inherent design of the open MRI system yields lower image quality and allows fewer imaging sequences than a classic clinical horizontal bore scanner and therefore may be less suited for the detailed characterisation of the raft properties.

The aim of this study was to determine the feasibility of characterising alginate forming raft properties using 3T supine MRI and visualisation of rafts in the open upright MRI system in a small‐scale healthy participant study.

## 2 Experimental

Some initial in vitro characterisations of the different formulations used were carried out to assess raft strength and chemical composition prior to the in vivo study. These methods are briefly described below.

### 2.1 Raft Strength

The raft strength of anti‐reflux suspension formulation was measured according to the British Pharmacopoeia procedure using a texture analyzer TA.XTplus with L‐shaped hook (A/ARH) from Stable Micro Systems. 10 mL and 20 mL of the anti‐reflux formulation were added to 150 mL of 0.1 M HCl. The formed raft was characterized via the L‐hook by the maximum force required to move the probe through the raft mass. Full details of the methodology can be found in the supplementary information, including Figure S1.

### 2.2 Forward (FW) Extrusion Process

Additionally, a second method was introduced by Dettmar et al. in 2005 [12]. The method itself is based on a forward (FW) extrusion process. This geometry is placed in a glass beaker filled with 150 mL of 0.1 M HCl. 10 mL of the formulation was added to the geometry. After 30 min, the HCl solution was decanted through the hole in the bottom of the geometry, and the formed raft was extruded through these holes via a piston attached to the Texture Analyzer. The work of extrusion is recorded according to the force over the distance to extrude the raft mass. Further methodology details are again given in the supplementary information, including Figure S2.

### 2.3 Chemical Composition

The materials were analysed according to ASTM F2259‐10 [13] via Nuclear Magnetic Resonance (NMR) spectroscopy using an AVANCE NEO 400 MHz spectrometer and a 9.4T magnet (Bruker Corporation). ^1^H NMR spectra were recorded using 90° pulse direct excitation of 10 μs, an acquisition time of 4 s and an additional recycle delay of 2 s between scans.

#### 2.3.1 Study Participants

Ethical approval for the study was obtained from the University of Nottingham Medical School Ethics Committee (ref 209‐0223) and all participants gave written informed consent. Healthy participants (aged between 18 and 55 years) were recruited to be scanned with either a 3T conventional scanner in the supine position or a 0.5T open scanner in the upright sitting position. Inclusion and exclusion criteria are given in the supplementary information.

#### 2.3.2 Study Day Drinks and Protocol

Participants attended for three separate MRI visits with a minimum 7‐day gap between sequential scans (max 21 days). They were given a different alginate product on each visit. The order of the alginate products was set using a Latin Square block design. Formulation details are given in Table 1.

**TABLE 1:** Formulation details of the alginate products commercially available and sourced from Turkey.

	Recommended dosage	Na‐Alginate (per recommended dose)	Na‐H‐carbonate (per recommended dose)	Ca‐carbonate (per recommended dose)
Formulation	mL	mg	mg	mg
X	10	500	267	160
Y	10	500	267	160
Z	10	500	267	160

*Note:* Manufacturer information: X: Gasvin (DEVA Holding, Turkey), Y: Gaviscon (Reckitt Benckiser Healthcare, UK), Z: Pronat (VEM Ilac San ve Tic A.S., Turkey).

Participants were asked to attend each session in the morning after an overnight fast from midnight the previous night (minimum 9 h fast). They were also asked to abstain from alcohol for the previous 24 h and caffeine and strenuous exercise for the previous 18 h. After reconfirming consent to participate and reassessment of MR safety questions, the participant was changed into disposable MRI‐compatible overalls.

Participants then had a 10‐min baseline scan to check that the stomach was empty prior to providing the interventions.

##### 2.3.2.1 3T Supine Scanner Only

The participant came out of the scanner and sat upright to consume the liquid drinks. The drink consisted of 200 mL Vanilla Fortisip (300 kCal preload) followed 20 min later by a 500 mL Lemon Juice drink (60% Water, 10% Sugar, 30% Lemon Juice, 246 kCal) [8]. This was followed immediately by 20 mL of the alginate formulation classed as a ‘maximum dose’ (Table 1 shows details for recommended 10 mL dose). The Fortisip drink switched the stomach from a fasted to fed state using a high calorie low volume drink and slowed the emptying of the stomach to enable the lower calorie higher volume acidic lemon juice drink to remain in the stomach during the study period.

##### 2.3.2.2 0.5T Upright Scanner Only

The participant stayed within the magnet and was asked to consume the same drink as the 3T scanner.

Immediately following consumption of the drinks and formulation the participant was then scanned using a variety of sequences to determine the position, volume and other characteristics of the alginate raft formed. Scans were then acquired every 20 min for approximately 2 h (six different time points).

#### 2.3.3 MRI Methods and Analyses

##### 2.3.3.1 3.0T Supine Scanner Only

###### 2.3.3.1.1 Acquisition

Participants were scanned supine in a 3.0T Widebore Ingenia Scanner (Philips, Best, The Netherlands). A 16‐channel DS Anterior coil was placed on the abdomen and used in combination with a 16‐channel posterior coil which was inside the scanner bed. The sequences used for the 3.0T scanning are given below and parameters are summarized in Table 2.

**TABLE 2:** Sequence parameters for 3T supine scanning.

Sequence	FOV mm^2^	Acq matrix (recon matrix)	Slice (gap) mm	No. slices	SENSE	FA°	Orient	Breath hold (s)	TR/TE ms
HASTE (Gast vol)	400 × 280	224 × 199 (400 × 400)	5 (1)	30–38	2.5	90	Axial	15–19	493/80
HASTE (Raft vol)	400 × 280	224 × 199 (400 × 400)	5 (1)	30–38	2.5	90	Acial	2 × 12–15	774/300
T2‐prep bTFE^a^	360 × 288	212 × 205 240 × 240	7 (0)	1	No SENSE	50	Axial	2 s per prep value, 15 s gap	3.6/1.8
HASTE (High res placement)	400 × 400	288 × 239 (400 × 400)	8 (0)	16–20	2.0	90	Cor	2 × 13 or 1 × 20	1222/80
HASTE (High res)	400 × 400	400 × 398 (400 × 400)	5 (0.5)	2	2.0	90	Cor	4	1878/80
HASTE (High res)	400 × 400	400 × 400 (400 × 400)	5 (0.5)	2	2.0	90	Cor	4	2091/160

Abbreviations: HASTE, Half‐fourier Acquisition Single‐shot Turbo spin‐Echo; vol, volume; res, resolution; FOV, Field of View; Acq, acquisition; recon, reconstructed; SENSE, SENSitivity Encoding; FA, Flip Angle; Orient, Image Orientation; TR = Repetition time; TE = Echo Time; Cor = coronal orientation.

^a^ T2prep values used were 20, 50, 80, 120, 180, 300, 500 ms.

###### 2.3.3.1.2 Gastric Emptying and Raft Volume

Axial T2‐Weighted (T2W) Half Fourier Acquisition Single‐shot Turbo Spin‐Echo (HASTE) sequence with 30–38 slices of 5 mm slice thickness, with a 1 mm gap between slices. The number of slices depended on the size of the stomach and the ability of the participant to hold their breath well. The echo time (TE) was 80 ms, which produced images that were moderately T2‐weighted. This sequence was acquired in a single breath hold of approximately 15–19 s.

###### 2.3.3.1.3 Raft Volume: Additional Scan

To aid in the visualization of the raft, a second Axial T2W HASTE scan was acquired with the same slice thickness, gap, and image field of view as the previous sequence, but with the echo time = 300 ms. This sequence produced images that were heavily T2‐weighted, with the more solid raft appearing very dark in the images and the liquid meal appearing very bright. Flow artifacts, which also appear dark in the images and which might be present in both this sequence and the previous sequence, were assumed to be random and not the same between the two sequences; however, the raft was assumed to be in the same location for both sequences. This was acquired in two breath holds of approximately 12–15 s each.

###### 2.3.3.1.4 T2 Measurements in the Raft

This sequence used a T2prepared‐balanced turbo field echo (T2prep‐bTFE) sequence [14] to acquire a single axial slice of 7 mm thickness through the raft in the stomach. It was repeated with seven different T2prep echo times (20, 50, 80, 120, 180, 300, 500 ms). Each different T2prep echo time image was acquired in a single breath hold with a 15 s gap between the acquisitions to allow for the system to return to equilibrium.

###### 2.3.3.1.5 Placement of High‐Resolution Scans Through Raft

To aid in the placement of some high resolution T2W scans through the raft, a set of lower resolution coronal T2W HASTE scans was acquired. Up to 20 slices of 8 mm slice thickness with no gap between slices were acquired through the whole stomach. These images were moderately T2W. The number of slices varied depending on the size of the stomach, and the data was acquired in either one (20s) or two (13 s) separate breath holds.

###### 2.3.3.1.6 High ‐Resolution Scans Through Raft

T2W HASTE scans were acquired in the coronal plane with a slice thickness of 5 mm and two slices with a 0.5 mm slice gap. Two different echo time scans were acquired to change the image contrast in the raft, TE = 80 ms and TE = 160 ms. These were acquired in two separate short breath holds of less than 5 s each.

###### 2.3.3.1.7 Analyses

Observers carrying out the analysis had more than 5 years of experience in analyzing gastro‐intestinal MRI data. All volumes were measured using the Medical Image Processing and Visualization (MIPAV version 11.0.8) software [15].

###### 2.3.3.1.8 Gastric Content and Air Volumes

These were measured by a single observer, by manually drawing around the content and air in the stomach on all slices which contained the organ. These regions of interest (ROIs) were then summed across all the slices to generate a total content or air volume in mL. This was carried out using the avial T2W images with echo time (TE) = 80 ms.

###### 2.3.3.1.9 Raft Volumes

These were measured by a single observer, by manually drawing around the raft in the stomach on all slices which visually contained the raft. To aid in the decision making of whether a particular slice through the stomach contained the raft, both the T2W axial image sets along with the lower resolution coronal T2W sequence were visualized.

###### 2.3.3.1.10 T2 of Raft

Small ROIs were drawn on the T2 datasets at different positions within the raft by a single observer. The mean signal intensity of the ROI at all the different T2prep TEs were used to calculate the T2 of the raft using an in‐house program which modelled the effect of the T2‐preparation scheme and subsequent imaging sequence [14]. ROIs in the liquid‐only region of the stomach were also drawn to compare the T2 calculated in the liquid with that of the gel raft.

###### 2.3.3.1.11 Image Texture Analysis of Raft

The high‐resolution coronal images showed that visually the rafts looked to have different signal contrast which can be described as different ‘texture’ in the image. To test whether this difference in ‘texture’ could be quantified, the high‐resolution coronal images with TE = 80 ms were put through an analysis pipeline to generate a numerical ‘texture’ value for the stomach region which was based on the contrast differences in the images using the Haralick ‘contrast’ algorithm [16]. This produces a z‐score which is a standardized output from the analysis. The ‘contrast’ parameter generates a higher score for regions which have large differences in pixel intensities and a low score for those with very similar pixel intensities. Further details of the process can be found in the supplementary information (including Figures S3 and S4). Image ‘texture’ in the raft on the T2‐weighted images will be generated from trapped gas and differences in raft gel strength and distribution.

##### 2.3.3.2 0.5T Scanner Only

###### 2.3.3.2.1 Acquisition

Participants were scanned upright (80° bed angle) in a 0.5T Open Paramed Scanner (ASG Superconductors, Genova, Italy). A body coil was placed around the abdomen and padding was added inside the coil to minimize motion of the abdomen. Arms were rested on a pillow at the top of the coil to keep them out of the imaging Field of View (FOV).

Various image sequences were used to visualize the raft in the stomach. However, at each time point, the following sequences were always acquired, and parameters are summarized in Table 3.

**TABLE 3:** Sequence parameters for 0.5T upright scanning.

Sequence	FOV mm^2^	Acq matrix (recon matrix)	Slice (gap) mm	No. slices	FA°	Orient	Breath hold (s)	TR/TE ms
FSE^a^	350 × 301	116 × 116 (173 × 114)	10 (3)	12 14	90	Cor	20 23	3294/102 3801/102
HASTE‐Sag	350 × 280	228 × 164 (318 × 229)	8 (4)	3 5	90	Sag	5 8	2432/120 3953/120
HASTE‐Ax	350 × 280	228 × 164 (318 × 229)	8 (1)	6 10	90	Axial	9 15	4459/64 7601/64
HASTE‐Ax	350 × 280	228 × 164 (318 × 229)	8 (1)	6 10	90	Axial	10 16	4865/120 7905/120
HASTE‐Ax	350 × 280	228 × 164 (318 × 229)	8 (1)	6 10	90	Axial	10 17	5270/180 8564/180

*Note:* For HASTE sequences, breath hold runs over two TRs.

Abbreviations: HASTE, Half‐fourier Acquisition Single‐shot Turbo spin‐Echo; vol, volume; res, resolution; FOV, Field of View; Acq, acquisition; recon, reconstructed; FA, Flip Angle; Orient, Image Orientation; TR, Repetition time; TE, Echo Time; Cor, coronal orientation; Sag, sagittal orientation.

^a^ Echo Train Length was 19. Six shots were used.

###### 2.3.3.2.2 T2W Fast‐Spin‐Echo (FSE) Sequence

This was a coronal sequence which covered the full stomach anatomy. It had a 10 mm slice thickness with a 3 mm slice gap. A total of 12–14 slices were acquired in a single approx. 20–23 s breath hold. This sequence had quite low image resolution and it was not always possible to visualize the raft. This sequence was used to measure the gastric content and air volumes.

###### 2.3.3.2.3 T2W HASTE Sequence in Sagittal Direction

This sequence was either 3 (initially for first subject first visit) or five slices of 8 mm slice thickness with 4 mm slice gap. This was positioned through the main body of the stomach to generate an image which cut through the raft to visualize the depth of the raft and for positioning of the axial scans. Good contrast between the meal and raft was generally seen with these images. The images were acquired in a 5 or 8 s breath hold.

###### 2.3.3.2.4 T2W HASTE Sequence in Axial Direction

This sequence was set up and positioned in the plane of the raft with a minimum of 6 slices; however, additional slices could be added if the raft appeared to cover a greater depth. This sequence was run with 3 different image contrasts using TE = 64, TE = 120, and TE = 180 ms. The slice thickness was 8 mm with a 1 mm gap. Breath holds were between 9 and 17 s.

###### 2.3.3.2.5 Analyses

Observers carrying out the analysis had more than 5 years of experience in analyzing gastro‐intestinal MRI data. All volumes were measured using the Medical Image Processing and Visualization (MIPAV version 11.0.8) software [15].

###### 2.3.3.2.6 Gastric Content and Air Volumes

These were measured using the same method as the 3T data, with the analysis carried out using the coronal FSE sequence, which covered the full stomach anatomy.

###### 2.3.3.2.7 Approximate Raft Volumes

It was not always possible to clearly visualize the bottom of the raft in the axial plane, and on some occasions, the bottom of the raft had not been included in the images acquired. Therefore, to gain an approximation of the raft volumes, the measurements of the approximate depth of the raft were carried out on the sagittal HASTE images, and the approximate cross‐section of the raft was defined from an axial slice in the center of the raft. These two measurements, made by a single observer, were then multiplied together to give an approximate raft volume—assuming an approximately cylindrical‐shaped mask. To check whether the data were consistently over or underestimating the volumes, the full raft volume was also measured from the axial images and compared to the approximate value for two to three datasets for each subject.

#### 2.3.4 Statistics

##### 2.3.4.1 In Vitro Data

Data are presented as median and inter‐quartile range (IQR), apart from the NMR spectroscopy data which is presented as median and range. A nonparametric one‐way ANOVA (Kruskal–Wallis) was carried out (using Prism version 10.2.3, GraphPad Software LLC) to determine whether there were differences in raft strength, raft mass, and extrusion strength for the same dose of the different formulations. Dunn's multiple comparison test (with adjusted *p*‐values) was then carried out to determine which formulations had significant differences.

##### 2.3.4.2 In Vivo Data

All analyses were conducted with the observer blind to which alginate formulation had been consumed. Data are presented as median and IQR. As this was a feasibility study with very small numbers of participants, no statistical tests were carried out. Comparison of data between upright and supine positions was carried out by pooling all data from the different formulations.

## 3 Results

### 3.1 In Vitro Characterisation of Raft

**FIGURE 1:**
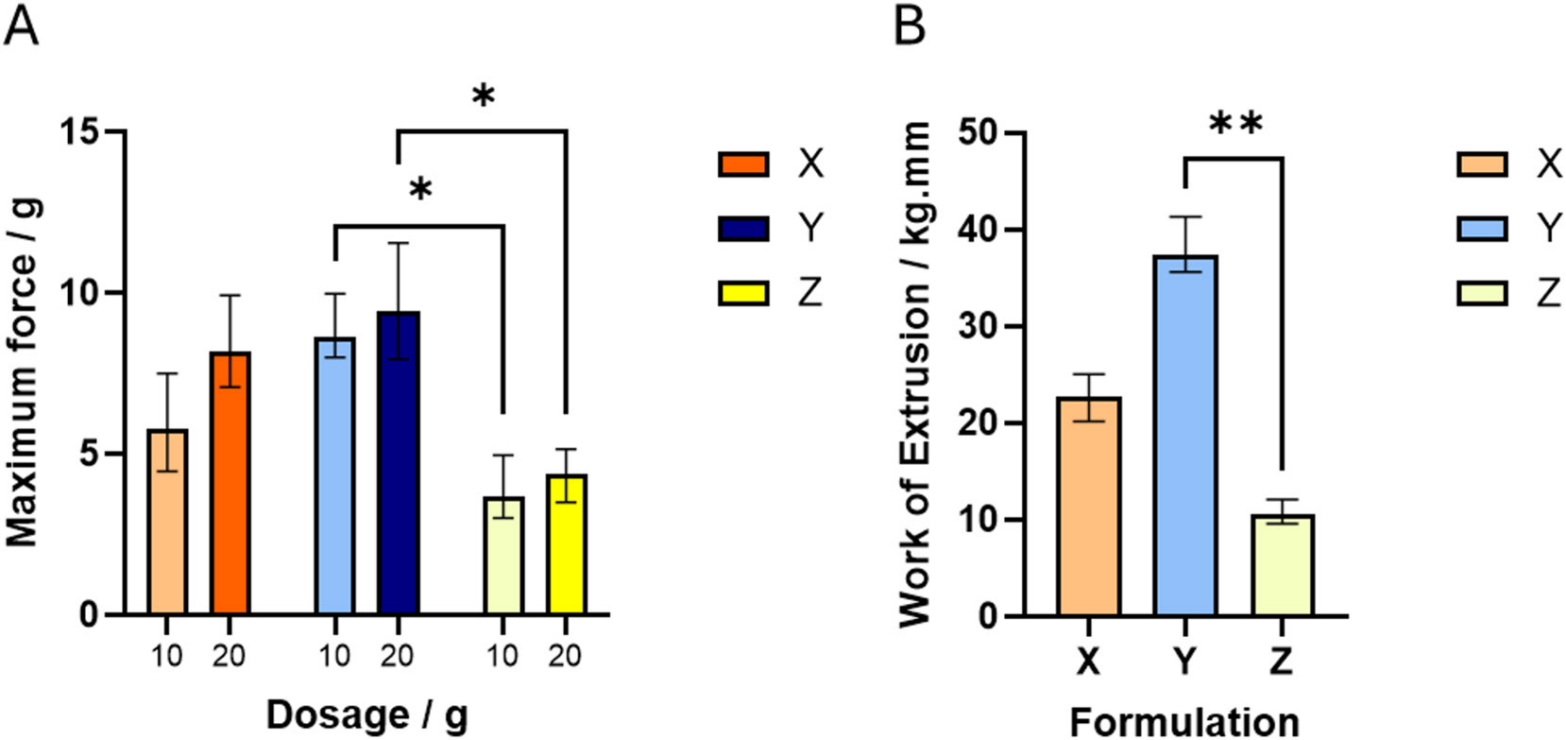
In vitro testing results. (A) Results from L‐hook raft strength experiments. (B) Results from the forward extrusion experiment (carried out only on a 10 g dose). Statistics shown on graphs from one‐way ANOVA (Kruskal–Wallis) between formulations, followed by Dunn's multiple comparison test with adjusted *p*‐values. *p < 0.05, **p < 0.01.

**FIGURE 2:**
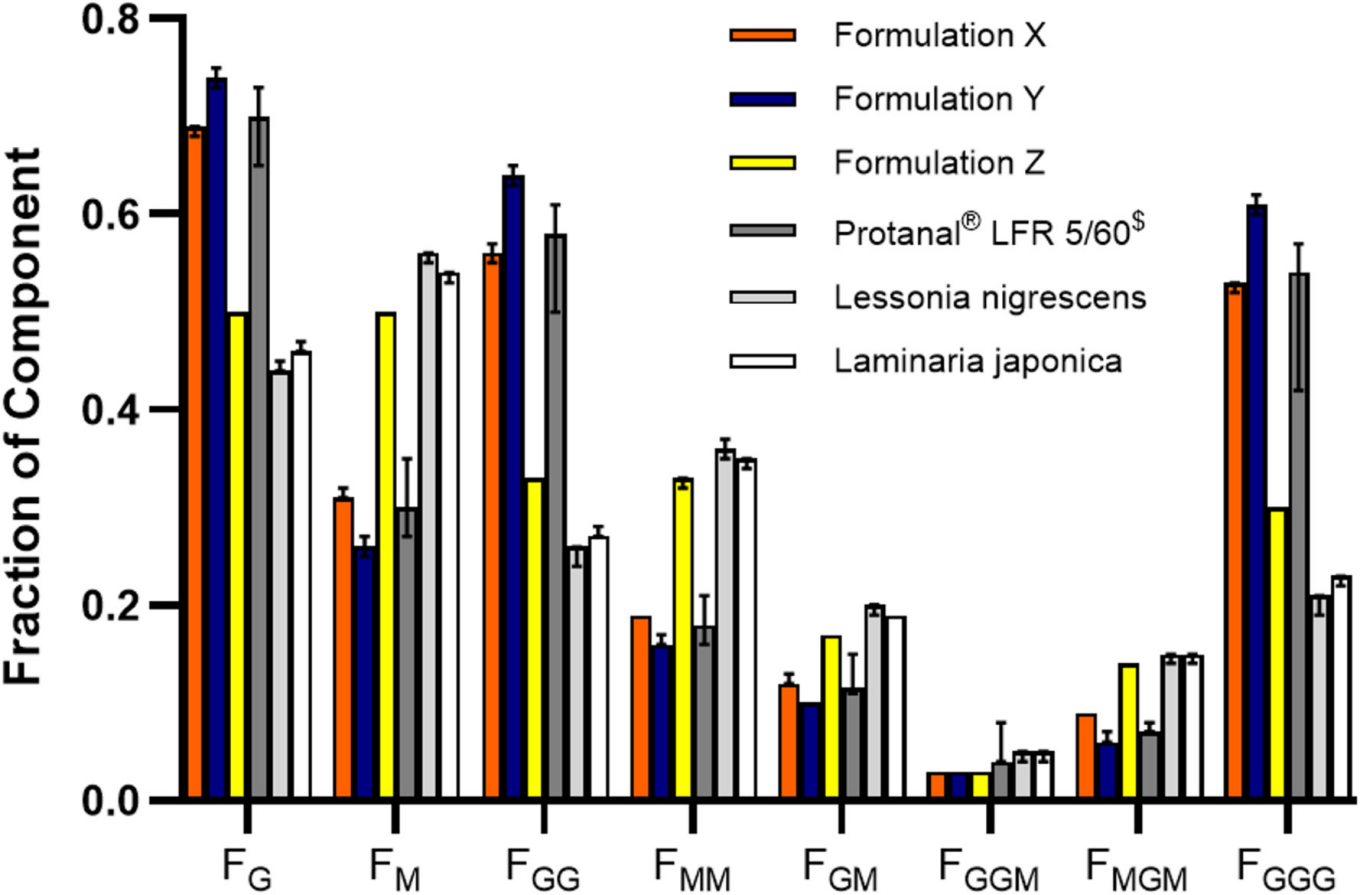
Graph showing NMR analysis of the components of the alginates isolated from the formulations along with the Protanal LFR 5/60 (obtained from Laminaria hyperborea) and two other sodium alginates obtained from other brown algae. F_G_—fraction of single guluronate units, F_M_—fraction of single mannuronate units; F_GG_, F_MM_, F_GM_—fraction of two G unit block, two M unit block or mixed GM unit block; F_GGM_, F_MGM_, F_GGG_—fraction of block containing two G units connected to one M unit, block containing alternating MGM units or block containing only three G units in a row.

The results of the characterization according to the raft strength, FW extrusion experiments are shown in Figure 1A,B, with the raft mass given in the supplementary information (Figure S5). The raft strength and the investigated raft mass formed from these formulations showed a strong dosage/volume dependence. The comparison of the different products according to the raft strength (L‐hook) showed lower values for formulation *Z* based on the statistical analysis, whereas the other two formulations *X* and *Y* showed similar attributes. The work of extrusion led to results with reduced variance within the same formulation samples and here formulations *X* and *Y* showed larger differences—although these were not statistically significant (probably due to the small sample size). The chemical composition of the alginates isolated from the formulations in comparison to four alginate products obtained from different brown seaweed species is given in Figure 2 showing wide variation in the composition.

### 3.2 In Vivo Studies

**FIGURE 3:**
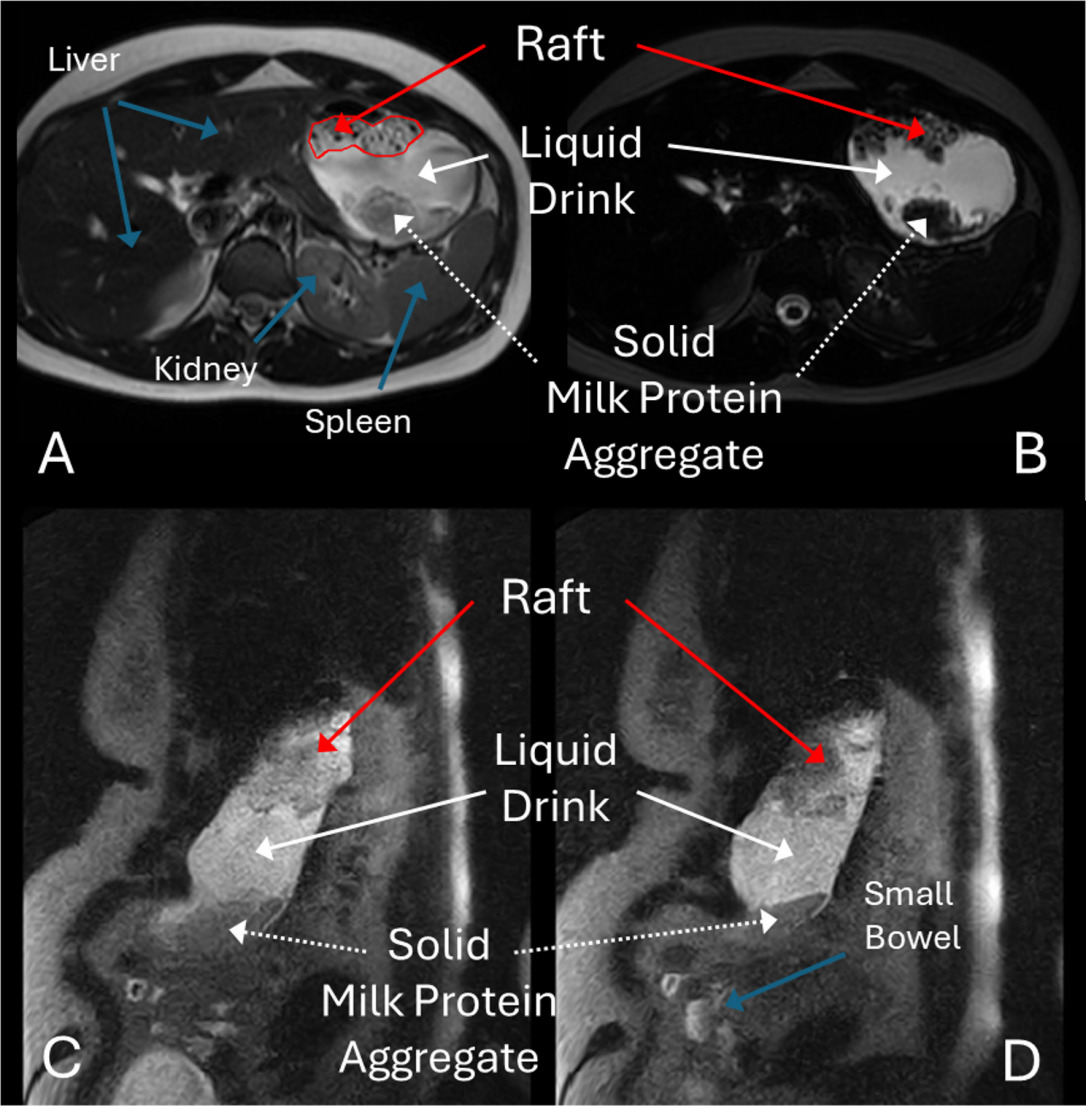
Images showing alginate raft in stomach. A&B Images from a participant at 3 T supine in the axial plane. (A) moderately T2‐weighted HASTE sequence, (B) heavily T2‐weighted HASTE sequence. (C, D) Images from a different participant 0.5 T upright in the sagittal plane. Images are consecutive slices from a moderately T2‐weighted HASTE sequence. The three components of the stomach contents are highlighted, alginate rafts—red arrows, liquid acid meal—solid white arrows, solid milk protein aggregate (from fortisip drink) ‐ dotted white arrows. Other relevant abdominal anatomy shown with blue arrows.

Six participants were recruited to the study (5 F, 1 M, median age: 28, range 23–50 years, median BMI 23.3, range 18.4–28.8 kg/m^2^). Three participants (3F) completed all studies supine at 3T and three participants (2F, 1 M) completed all studies upright at 0.5T. All participants consumed all study drinks and alginate formulations. Example images from both scanners showing the different components of the drinks consumed are shown in Figure 3.

#### 3.2.1 Volumes

**FIGURE 4:**
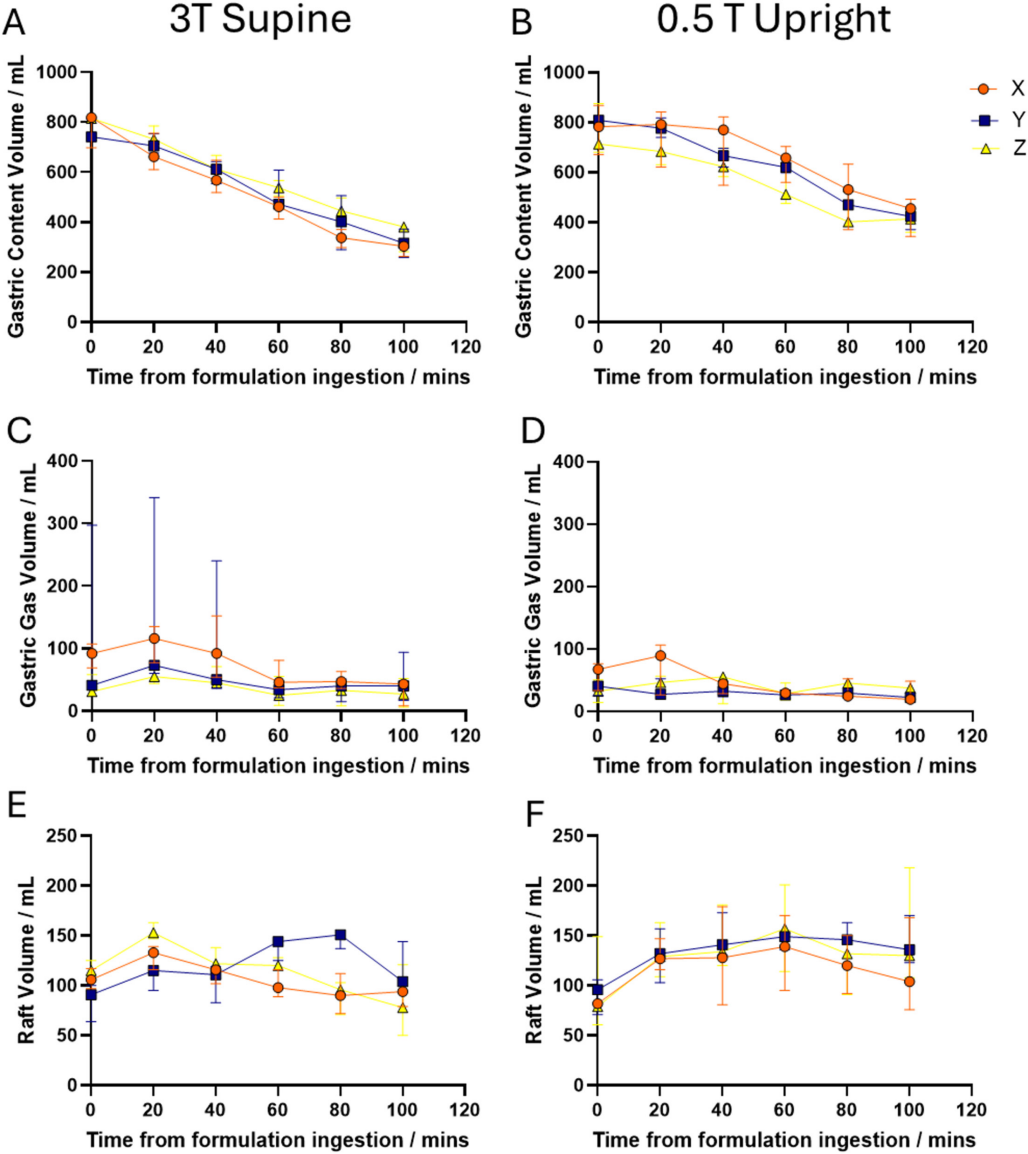
Graphs showing gastric content (A, B), gas (C, D) and raft volumes (E, F), split by formulation and scanner. Data are shown as median and range. *N* = 3 for each meal.

Figure 4 shows the gastric content volume data for the 3 different alginate products supine at 3T (Figure 4A) and upright at 0.5T (Figure 4B). This data shows the study drinks set up very similar gastric emptying conditions in the stomach across all study days. Gastric gas was generally variable across the study day with large variations across the subjects and formulations, Figure 4C,D. The raft volume data for the different formulations are shown in Figure 4E,F, showing larger variations between formulations supine at 3T compared to upright at 0.5T. The data showing how accurate the approximate raft volumes estimates were compared to fully drawn rafts can be seen in figure S6.

**FIGURE 5:**
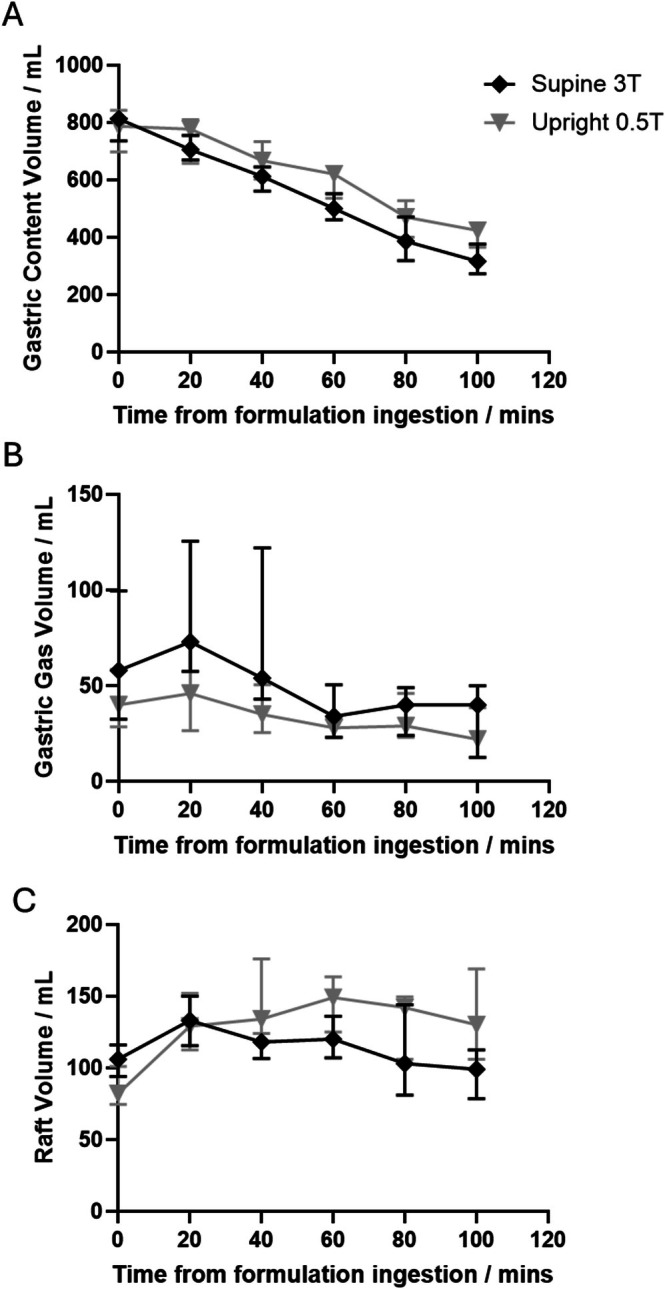
Graphs showing gastric content (A), gas (B), and raft volume (C) for the different scanner and body positions. Data pooled across all formulations. Data shown as median with error bars 95% confidence interval for median. *N* = 9 for each body position.

When the data from all formulations were pooled for each scanner, there appears to be slight differences in behaviour of the different volumes measured (Figure 5). The upright scanner appeared to retain a slightly larger volume for the total content of the stomach despite a similar initial volume (T0 data) (Figure 5A). There is generally less air in the upright stomach (Figure 5B) and the raft volume appears to increase for both scanners initially, but then decreases for the 3T supine position whilst remaining higher in the upright position (Figure 5C).

#### 3.2.2 Quantitative T2

Table 4 summarizes the data acquired from this measurement. Only two participants produced images of a quality acceptable for further analysis; one participant consistently produced images with large‐scale artifacts across the region of interest, and this data could not be analyzed. Data in the upper raft regions tended to have a lower T2 value compared to the lower regions. Table S1 summarizes the individual participant data.

**TABLE 4:** T2 data (median (range)) combined from two participants for the upper and lower regions of the raft. Where no data was measured either due to artefacts or lack of raft to draw ROI the data is left blank.

Time (mins)	Upper RAFT T2 data (ms)					
	Formulation *X*	*N*	Formulation *Y*	*N*	Formulation *Z*	*N*
0	49 (39–86)	6	55 (44–85)	6	148 (88–175)	3
20	50 (21–91)	5	72 (45–95)	5	89 (46–113)	6
40	33 (22–58)	5	85 (50–148)	5	98 (50–152)	6
60	87 (42–182)	6	57 (37–82)	6	75 (49–105)	6
80	69 (54–79)	6	57 (30–115)	6	75 (60–76)	3
100	73 (52–87)	6	46 (39–93)	6	83 (56–121)	6
	Lower RAFT T2 data (s)					
40		0	111 (109–114)	2	79	1
60	90 (84–95)	2	107 (73–104)	2	212 (188–236)	2
80	97 (91–127)	3	121 (89–153)	4		0
100	112 (87–125)	3	99 (78–133)	4	109	1

#### 3.2.3 Texture Analysis

**FIGURE 6:**
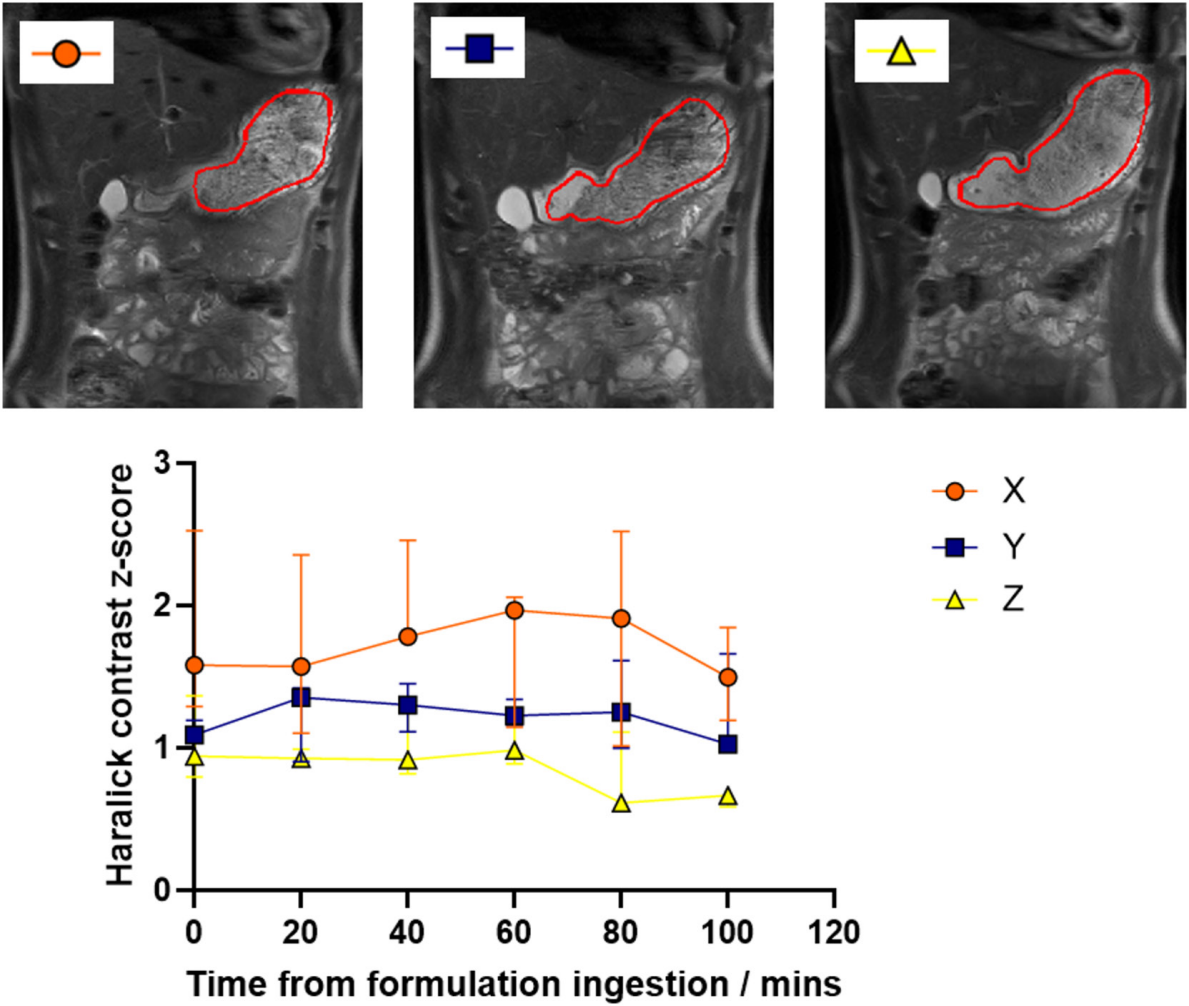
An example set of images from one volunteer at T40 min for the three different formulations. ROIs for the stomach used in the texture analysis shown in Red. Graph of Haralick contrast texture with time and formulation shown below images.

The texture analysis data and example images are shown in Figure 6. Although there is limited data from three participants, the haralick texture analysis shows that formulation X produced images with the highest contrast across the raft and formulation Z the lowest contrast.

**FIGURE 7:**
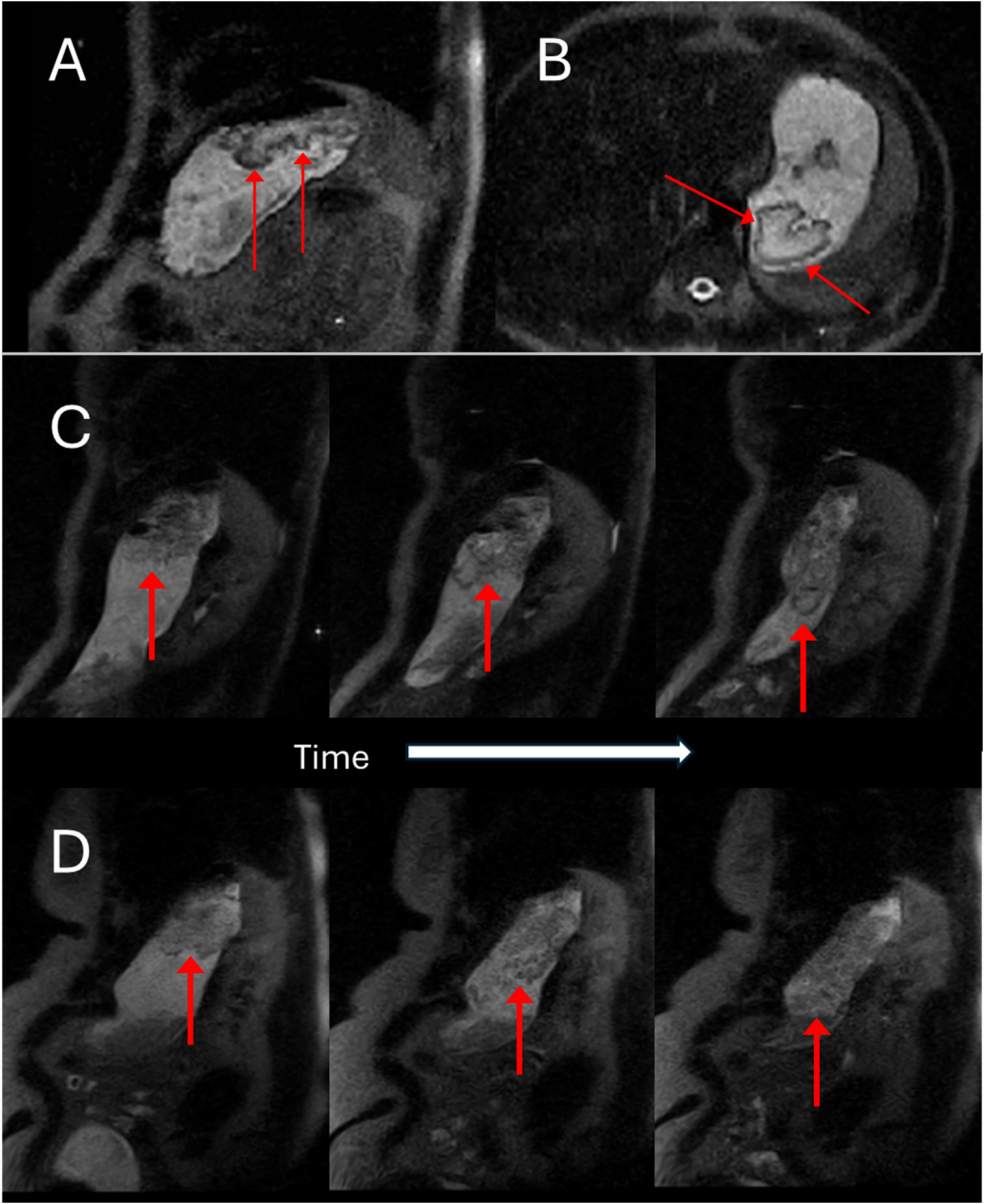
Images from the 0.5 T Upright scanner. Images A (sagittal) and B (axial) are from the same participant at the final T100 time point and illustrate the darker ‘halo’ effect of stronger gel encapsulating either ungelled or weaker gelled formulation. Figures C and D are from two different participants (and different formulations) whose stomach geometry allowed for the bottom of the raft to elongate in the head‐foot direction as the stomach emptied and the axial cross‐section of the stomach reduced. The sagittal images shown are from different time point in the study.

Some notable features captured from the upright scanner images are shown in Figure 7. In the later time points for one participant a darker ‘halo’ can be seen at the bottom edges of the raft (Figure 7A,B). For two participants the stomach geometry was elongated in the head‐foot direction, and this allowed for the bottom of the raft position to change substantially as the stomach contents emptied (Figure 7C,D).

## 4 Discussion

This was a small‐scale feasibility study which showed formulations which produced alginate rafts floating in the stomach could be visualised using both conventional supine scanning (at 3T), but also lower field (0.5T) open geometry, allowing for upright positioning of the participants. The study protocol produced a stomach that emptied at a very similar rate across different study days and allowed all formulations to produce rafts in all volunteers. This study builds on prior work [8, 9] which have investigated alginate rafts using supine MRI at lower field strength.

The in vitro study of the raft strength (L‐hook) of the three formulations showed statistical reduced values for product *Z*, whereas product *X* and *Y* showed similar performance. The alternative work of extrusion method showed reduced standard deviation and could further differentiate product *X* and *Y*. Here the highest values were detected for formulation *Y*. The chemical structure of the alginates extracted from the three formulations in comparison to four alginate products obtained from different brown seaweed species explained the reason for the difference in performance.

The alginates isolated from formulations *Y* had similar chemical structural elements as Protanal LFR 5/60 with high α‐l‐guluronic (G) acid content. These high G contents in the alginates are able to develop an egg‐box structure in combination with Ca^2+^ leading to strong gels [17]. These high G contents are a characteristic of Protanal LFR 5/60 obtained from Laminaria hyperborea.

The alginate isolated from formulation X showed reduced G contents and enhanced 1,4‐β‐D mannuronic (M) acid content, whereas for the alginate from formulation Z, even higher M contents were detected. The amounts of these chemical structural elements are similar in formulation Z to other alginate products obtained from different brown seaweed species. This shows the importance of the chemical structure of the alginates for the performance in anti‐reflux formulations, especially when the formulations are based on similar recipes (constant CaCO_3_ and NaHCO_3_ contents) as investigated here, with the ratio of G to M units shown to be important in determining the raft performance in a previous study [18]. The formulations of *X* and *Y* contained higher G units compared to Z. Such high G content alginates are able to form more junction zones with the Ca^2+^ ions, whereas high M content alginate networks form more entangled polymer structures in solutions. The high G content alginate junction zones are able to absorb and bind more water molecules, and therefore, the detected raft mass is enhanced, as seen for formulations *X* and Y compared to Z.

A previous study looked at the chemical effects of neutralisation of different formulations and showed wide differences between products [19] although these had differing amounts of alginate content in the formulations.

Raft behavior appeared to be different depending on body position, supine and upright. Specifically, raft volumes in the upright scanner did not decrease towards the end of the study period, unlike the raft volumes in the horizontal scanner, which decreased. These differences are probably due to the position of the raft within the stomach, with the supine position allowing the raft to float near the antral area and could more easily be broken down and emptied from the stomach. In this study, there also appeared to be a very slight difference in gastric emptying rate between the body positions, with the upright position emptying slightly slower. This may have been due to layering of the milk protein from the Fortisip drink after acidification in the stomach [20, 21], although some of the differences later in the study may have come from differences in the raft volume. The protein layer was positioned closer to the antrum in the upright position and may have caused a change in response in gastric emptying. Both body positions are relevant to the use of alginate anti‐reflux formulations, as some patients will consume the formulation after eating and others before they go to sleep. Therefore, knowledge of the evolution of the raft in both postures is of interest. Differences in body position have been investigated previously using continuous pH monitoring [22]. Results from the study showed that alginic acid formulations were equally effective compared to antacids for acid‐neutralization capacity only in the upright position and were not as effective when lying down. MRI may therefore be able to help understand the mechanisms of these observations.

The differences seen between upright and supine may also be attributed to the differences in image acquisition between the two scanners, as there would be larger partial volume effects from the upright scanning, which had a slice thickness of 10 mm and a gap of 3 mm compared to the thinner slices of 5 mm and a gap 1 mm supine at 3.0T. However, partial volume effects would be present for all measurements, and the differences in volume decreases occur after the initial postprandial volume, which was very similar between the two scanners. It was not possible to achieve a smaller slice thickness and gap and cover the full stomach in a single breath‐hold on the upright, and there is also the possibility that the differences come from the different individuals scanned due to intersubject variations. Therefore, further studies that can image the same individual both upright and supine are needed to understand if the emptying rates are different with the different body positions.

Quantitative measurements of T2 were carried out in two out of the three participants at 3T and showed that the raft formed was very heterogeneous. The area of the raft nearest the air, floating at the very top produced a shorter T2 value indicating a stronger gel, or more viscous formulation, compared to any lower regions that could be measured which may have been weaker or less viscous. Previous studies have also characterised gel in the stomach from various different food‐grade polymers [23, 24, 25, 26]. One study [23] showed trends of shorter T2 in alginate gels in vivo, which had been tested in vitro to be stronger gelling compared to the higher T2 in those with weaker gelling [10]. Another in vitro study has shown spatial dependence of T2 within the gelled structure [11]. The ‘halo’ effect which was seen at the bottom of one of the rafts in the upright scanner may indicate that a much stronger gel has been formed at the bottom of the raft and may be encapsulating ungelled formulation, or a much weaker gel [24] above it. Further studies are needed to understand whether this is a feature that can be reproduced.

The image texture analysis data produced a quantified parameter which correlated well with the visual appearance of the rafts in the high‐resolution images. Formulation *X* had the largest contrast in the rafts which probably relates to an increase in the amount of trapped gas within the raft for this product. Formulation *Z* had the lowest value reflecting the more homogeneous appearance of this raft. Other studies have looked to quantify image ‘texture’ of the stomach contents from feeding experiments and provide additional quantitative data to compare between study conditions [27, 28].

There were limitations to this study. Due to the small number of participants, no statistical testing of differences between the alginate formulations were carried out and all data could be considered as semi‐quantitative only. A larger study would be needed to ascertain whether the different raft strengths from the formulations produced significantly different measurements over time. For the supine scanning, there were no time restrictions placed as to when participants could use the toilet facilities, and this resulted in body position changes which were not consistent between subjects and across the different formulations. These changes may have influenced the volume of the rafts at 3T, and future studies would look to set a formal break time in the scanning. In addition, any changes to the position of the diaphragm during the two breath‐hold scan may have resulted in either a slight over or underestimation of the raft volume, but this should not have been significant as the raft covered many slices in the axial plane. Problems with banding artefacts from the balanced TFE image acquisition and motion of the stomach, both from respiratory position and stomach walls for the measurements of T2, yielded a reduced amount of data for this particular parameter. Images sets with just a few banding artefacts across the data could be used for quantifying the T2 in some locations within the raft, however those with many artefacts had to be completely discarded. The range of T2prep echo times allowed for some loss of individual echo time data, but still allowed for the T2 fitting calculation to be made. Future studies could look to use alternative imaging sequences to measure the T2 [29] to remove the banding artefacts and change the orientation to reduce the respiratory artefacts. In addition, repeated scans or extra T2prepartion echo times could be used to improve the measurements.

In conclusion, this small‐scale feasibility study showed it was possible to image alginate rafts formed in the stomach using both 3T supine and 0.5T upright MRI scanners. Quantification of different raft properties in vivo were carried out, on three alginate formulations which produced very different raft strength and mass from in vitro experiments, due to structural chemical differences in the alginates used. The data from this study suggested that the raft behaviour (emptying and breakdown) may differ when participants are supine compared to sitting upright after dosing. Further larger scale in vivo studies are needed to determine what differences to the raft characteristics in vivo can be determined from MRI (e.g., strength, volume, retention) from the different formulations and whether statistical differences in emptying rates from body positioning are found.

## Conflicts of Interest

Matthias Knarr is an employee of PS Biopolymers (previously known as IFF N&H, now part of ROQUETTE Health & Pharma Solutions). All other authors declare no competing interests in this work.

## Supporting information


**Figure S1.** Illustration of the method for raft strength testing with the L‐hook.
Figure S2 Illustration of the method for raft strength testing using the extrusion method.
Figure S3 Image showing map of z‐score following Haralick texture analysis contrast algorithm. Whiter regions in the map have a higher z‐score indicating a more heterogeneous contrast in the original image.
Figure S4 Definition of the stomach region for z‐score analysis. A: Original high‐resolution coronal image showing the liver (red) and stomach (orange) ROIs defined. Note the stomach ROI is inside the walls of the stomach. B: Z‐score map following Haralick texture analysis showing the stomach ROI. This ROI is inside the stomach walls to make sure the average z‐score from the stomach comes from the raft texture and not from any edges of the stomach which also have high z‐scores.
**Figure S5.** In vitro testing results of raft mass generated during L‐hook experiment. Statistics shown from 1 way ANOVA (Kruskal–Wallis) between formulations, followed by Dunn’s multiple comparison test with adjusted *p*‐values. ** *p* < 0.01.
**Figure S6.** Estimated volume of raft vs measured volume of raft for the three different participants. Line of identity is shown in black.
**Table S1.** Mean and standard deviation T2 quantitative measurements (ms) in the alginate raft formulations, split by formulation, volunteer and position. All data shown in seconds.

## Data Availability

The data underlying this article are owned by Roquette. Data will be shared on reasonable request to the corresponding author with permission of Roquette.
