# Supplementary material for: Investigations of Anti‐Reflux Formulations Containing Alginates Using MRI: A Feasibility Study Using Conventional 3.0T and 0.5T Open Upright Scanning
Source: NMR Biomed. 2025 Jul 10;38(8):e70090. doi: 10.1002/nbm.70090 (PMC12242899; doi:10.1002/nbm.70090)
Supplement: Supplementary file 1 — Figure S1. Illustration of the method for raft strength testing with the L‐hook. Figure S2 Illustration of the method for raft strength testing using the extrusion method. Figure S3 Image showing map of z‐score following Haralick texture analysis contrast algorithm. Whiter regions in the map have a higher z‐score indicating a more heterogeneous contrast in the original image. Figure S4 Definition of the stomach region for z‐score analysis. A: Original high‐resolution coronal image showing the liver (red) and stomach (orange) ROIs defined. Note the stomach ROI is inside the walls of the stomach. B: Z‐score map following Haralick texture analysis showing the stomach ROI. This ROI is inside the stomach walls to make sure the average z‐score from the stomach comes from the raft texture and not from any edges of the stomach which also have high z‐scores. Figure S5. In vitro testing results of raft mass generated during L‐hook experiment. Statistics shown from 1 way ANOVA (Kruskal–Wallis) between formulations, followed by Dunn’s multiple comparison test with adjusted p‐values. ** p < 0.01. Figure S6. Estimated volume of raft vs measured volume of raft for the three different participants. Line of identity is shown in black. Table S1. Mean and standard deviation T2 quantitative measurements (ms) in the alginate raft formulations, split by formulation, volunteer and position. All data shown in seconds. [file NBM-38-e70090-s001.docx]

Supplementary Information

Methods:

*In-Vitro Characterisation of formulations according to raft strength (L-hook)*

The method involved addition of the liquid formulation to 150 mL of 0.1 M Hydrochloric Acid (HCl) maintained at 37 ± 0.5 °C in a 250 mL low-form glass beaker. Once the acid had reached the set temperature, the formulation was added with a syringe in a dosage level of 10 or 20 mL. The raft formed around an L-shaped stainless-steel wire probe held upright in the beaker throughout the whole period of raft development. After 30 min, the beaker with the raft was transferred to the Texture Analyzer platform and the L-shaped hook was attached to the probe adapter of the Texture Analyzer. A 1 kg load cell was used in this experiment. The probe was vertically lifted upward through the raft at a speed of 5 mm/s while the force is measured in tension mode.

The maximum force (in g) required to move the L-hook through the sample is measured by the load cell. The specification limit for raft strength as per British Pharmacopoeia is not less than 7.5 grams.

Figure S1. Illustration of the method for raft strength testing with the L-hook.

*In-Vitro Characterisation of Formulations according to work of extrusion*

The FW extrusion cell described in this method was placed into a 400 mL high-form glass beaker. After 150 mL of pre-heated 0.1 M HCl (37 ± 0.5 °C) had been filled into the glass, a dose of 10 mL of the anti-reflux formulation was added (corresponding to approx. 500 mg of sodium alginate). This beaker was then stored for 30 min at 37 °C. Afterwards the HCl solution was decanted through the holes in the bottom plate and the raft gel remained in the geometry, from where the gel is extruded through the openings in the bottom plate by a piston connected to the Texture Analyzer. A 5kg load cell was used in this experiment. The required force was recorded during the measurement where the gel was extruded through the honeycomb structure in the bottom plate. See figure S2 for an illustration of the method.

Figure S2 Illustration of the method for raft strength testing using the extrusion method.

*Study Participant Inclusion and Exclusion Criteria:*

Inclusion Criteria

- Healthy: healthy status will be confirmed at screening using a medical questionnaire
- Participant is willing and able to give informed consent for participation in the study
- Not currently taking any regular medications
- BMI < 30 kg/m^2^

Exclusion Criteria

- nability to complete the informed consent process and/or MRI safety questionnaire.
- Known contraindication to MRI scanning (for example, pacemaker/implanted defibrillator, aneurysm clips, implanted programmable device, intra-ocular metallic fragment, claustrophobia, etc.).
- Major psychiatric, cognitive or mood disorder.
- Any other significant chronic medical conditions, including diabetes (type 1 and 2), asthma and epilepsy and IBD.
- Any acute major illness or surgery within one year prior to participation.
- Someone who smokes, vapes or uses nicotine patches
- Implanted metalwork in the torso (close to the stomach) which would degrade the MR image quality.
- Inability to lie flat on their back for several hours.
- Having taken part in a research study in the last 3 months involving invasive procedures, ionising radiation or an inconvenience allowance.

*Image Texture Analysis*

Image normalisation: To make sure that the image contrast only came from differences in the raft and not from differences in the scanner settings on different days the images had to be normalised. The liver tissue signal was used for this process as it was always visible in the images at every visit and every time point and should not change its MRI properties for the duration of the study. A region of interest was drawn in the liver avoiding large blood vessels which could appear either very bright (white) or very dark (black). The median signal intensity from the liver was then used to normalise the images by generating a scaling factor for the image set to be multiplied by.

Haralick Algorithm Contrast Image Generation. The Haralick algorithm uses intensity information on small scale regions within the image to generate scores on different properties of these intensities. For this study a 5 x 5 pixel region was chosen with no directional preferences (i.e. all directions were calculated and averaged together) and the contrast parameter was calculated. This means that regions that have large variation in their pixel intensities over the 5x5 area have a high score and those that have very little variation have a low score; the output from these measurements are standardised into a z-score. This calculation is carried out across the whole image to generate a map – Figure S3.


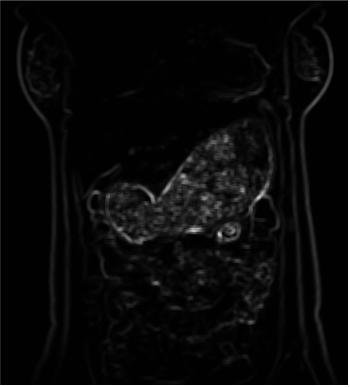


Figure S3 Image showing map of z-score following Haralick texture analysis contrast algorithm. Whiter regions in the map have a higher z-score indicating a more heterogeneous contrast in the original image.

Z-Score Output. The stomach region was defined on the high-resolution image with the ROI set inside the stomach wall to make sure no ‘texture’ from the wall was included in the ROI (Figure S4). The mean and standard deviation of the z-score was then calculated from the texture map.


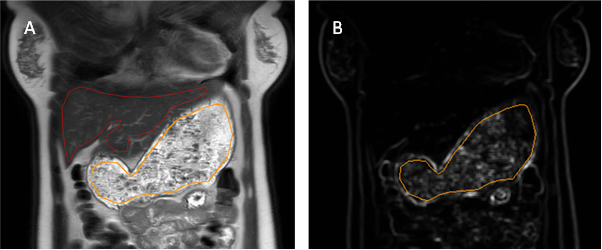


Figure S4 Definition of the stomach region for z-score analysis. A: Original high-resolution coronal image showing the liver (red) and stomach (orange) ROIs defined. Note the stomach ROI is inside the walls of the stomach. B: Z-score map following Haralick texture analysis showing the stomach ROI. This ROI is inside the stomach walls to make sure the average z-score from the stomach comes from the raft texture and not from any edges of the stomach which also have high z-scores.

Results:

*In-Vitro Study Results*

Figure S5 shows the mass data from the L-hook experiment, which again shows the significant differences between formulations Y and Z for both dosages used. Formulation Y producing a larger raft (in terms of mass) compared to Z.

Figure S5. In-vitro testing results of raft mass generated during L-hook experiment. Statistics shown from 1 way ANOVA (Kruskal-Wallis) between formulations, followed by Dunn’s multiple comparison test with adjusted p-values. ** p< 0.01.

*In-Vivo Study Results*

Figure S6 shows the relationship between estimated and measured raft volumes for the 3 subjects from the 0.5 T Upright scanner. The estimated method tended to slightly underestimate the total raft volume across the wide range of volumes measured.

Figure S6. Estimated volume of raft vs measured volume of raft for the 3 different participants. Line of identity is shown in black.

Table S1 showed the individual data results for the quantitative T2 measurements in the rafts showing large variability across the subjects and formulations.

Table S1. Mean and standard deviation T2 quantitative measurements (ms) in the alginate raft formulations, split by formulation, volunteer and position. All data shown in seconds.

Unless otherwise stated measurement average calculated from 3 separate regions across the raft.

| Time from formulation ingestion (mins) | Formulation X | Formulation Y | Formulation Z |
| --- | --- | --- | --- |
|  | Upper Regions of Raft (ms) | | |
| 0 | 65±20 | 61±21 | No data  – stomach moving |
|  | 44±6 | 60±9 | 137±45 |
| 20 | 21±0§ | 71±25 | 78±33 |
|  | 99±54 | 74±13§ | 91±27 |
| 40 | 37±19 | 116±46§ | 103±51 |
|  | 44±16§ | 79±25 | 103±40 |
| 60 | 110±70 | 50±12 | 67±16 |
|  | 79±21 | 71±15 | 84±18 |
| 80 | 64±9 | 44±14 | 70±9 |
|  | 71±11 | 82±30 | No data  – stomach moving |
| 100 | 77±8 | 44±4 | 74±16 |
|  | 64±19 | 66±26 | 95±26 |
|  | Lower Regions of Raft (ms) | | |
| 40 | No ROI drawn | No ROI drawn | No ROI drawn |
|  | No ROI drawn | 109±4§ | 79* |
| 60 | 95* | 107±47§ | No ROI drawn |
|  | 84* | No ROI drawn | 212±34§ |
| 80 | 112±21§ | 121±4§ | No ROI drawn |
|  | 91* | 121±45§ | No data  – stomach moving |
| 100 | 119±9§ | 105±39§ | No ROI drawn |
|  | 87* | 99±3§ | 109* |

Volunteer 1 (Black on white), Volunteer 2 (Black on grey). § Data from only 2 measurements not 3. * Only a single measurement made therefore no standard deviation could be calculated.
